# The role of caregivers in the clinical pathway of patients newly diagnosed with breast and prostate cancer: A study protocol

**DOI:** 10.3389/fpsyg.2022.962634

**Published:** 2022-11-02

**Authors:** Clizia Cincidda, Serena Oliveri, Virginia Sanchini, Gabriella Pravettoni

**Affiliations:** ^1^Applied Research Division for Cognitive and Psychological Science, European Institute of Oncology IRCCS, Milan, Italy; ^2^Department of Oncology and Hemato-Oncology, University of Milan, Milan, Italy; ^3^Department of Public Health and Primary Care, Centre for Biomedical Ethics and Law, KU Leuven, Leuven, Belgium

**Keywords:** caregiver, breast cancer, prostate cancer, psychological intervention, adherence

## Abstract

**Background:**

Caregivers may play a fundamental role in the clinical pathway of cancer patients. They provide emotional, informational, and functional support as well as practical assistance, and they might help mediate the interaction and communication with the oncologists when care options are discussed, or decisions are made. Little is known about the impact of dyadic dynamics on patient-doctor communication, patient's satisfaction, or adherence to the therapies. This study protocol aims to evaluate the efficacy of a psychological support intervention on patients-caregivers relationship and their alignment in the treatment decision-making (TDM) process and estimate related improvement in patient' compliance/adherence to treatments.

**Methods:**

A total of 102 patients-caregivers' dyads will be involved, among breast and prostate cancer patients. The study entails a pre- post- evaluation through psychological questionnaires, with a randomization of participants in two conditions, the experimental one in which subjects participate in a psychological support consultation, and the control one, where dyads do not receive any intervention. A follow up after 6 months from the enrollment is planned.

**Discussion:**

A positive impact of the psychological support intervention on patients' anxiety, depression, distress, and perceived social support is expected. Such improvements can directly affect patients' satisfaction and adherence to treatments. Data gathered from this study may inform health care providers, policy makers, and public health managers about the importance of caregiver's involvement in the cancer care pathway, and the best way to manage it. A further *impact* is to develop a specific intervention protocol to support caregivers' involvement in cancer care pathway, improve patient's wellbeing, the interaction with physicians and the compliance with the cancer treatment.

## Introduction

Caregivers' involvement in the patients' cancer care path and in the decision-making process has become more frequent in the last decades and has been recognized by a growing empirical literature, according to which most patients and caregivers preferred a collaborative or supportive caregivers' involvement (Laidsaar-Powell et al., 2017). Throughout the treatment process, from diagnostic assessment onwards, oncological patients commonly face the dramatic experience of cancer with at least one caregiver at their side, deal with visits and treatment appointments, therapies' side effects, change in daily life, experiencing significant distress (Laidsaar-Powell et al., 2017; Laryionava et al., 2018; Fatigante et al., 2021). Caregivers generally belong to the family system, often they are the spouse/partner or the adult child of the patients, in rare cases even friends can play this role (Laidsaar-Powell et al., 2017), They provide emotional, informational, and functional support as well as practical assistance, also mediating the interaction and communication with the oncologists when care options are discussed, or decisions are made (Hobbs et al., 2015; Litzelman, 2019; Schulman-Green et al., 2020; Fatigante et al., 2021).

Laidsaar-Powell et al. (2017) developed a conceptual framework (TRIO) according to which caregivers are involved in various ways in the decision-making process, concerning primarily the daily management of cancer treatments' effect. Patients almost always involve caregivers in the decision-making process, transforming the classic patient-physician interaction into a triadic relationship (patient, caregiver, and physician) (Mitnick et al., 2010; Renzi et al., 2016; LeBlanc et al., 2018). Nevertheless, patients and caregivers may have different perspectives regarding treatment decisions, experiencing episodes of conflicts or tensions between them, especially in the advanced cancer and the end-of-life context (Levine and Zuckerman, 1999, 2000; Vivian, 2006; Kramer et al., 2010; Hauke et al., 2011; Boelk and Kramer, 2012; Shin et al., 2016; Longacre et al., 2018; Benson et al., 2019; Hansen and Tjørnhøj-Thomsen, 2020). Such disagreements may have a negative impact on the patient, affecting the understanding of medical information, compliance with therapies and, consequently, the patient's quality of life, including the relationship with the caregiver (Shin et al., 2016). Poor disease-related communication between cancer patients and caregivers could increase the levels of psychological distress for both parties, making the course of treatment more complex (Speice et al., 2000). Instead, when patients and their caregivers are in line with the decisions to be made and their preferences are met, the process of care is enhanced for all the parties involved (Speice et al., 2000; Joosten et al., 2008). Moreover, caregivers' involvement in the oncological examinations was associated with both increased patients' satisfaction with respect to the care process and improved understanding of cancer-related information (Joosten et al., 2008; DuBenske et al., 2010a,b). More broadly, Krieger (2014) suggests the importance of an alignment among patient-caregiver preferences for the extent of caregiver involvement in the decision-making process.

The issue of caregivers' involvement in the therapeutic path has been also conceptualized within the ethical and bioethical literature. Within this literature, caregivers' involvement has been discussed in relation to the concept of autonomy. While some authors promoted an interpretation of autonomy as self-determination (Beauchamp and Childress, 2019), thus suggesting that the caregivers have only a supportive role (Mitnick et al., 2010), the vast majority of contemporary contributions on the topic tend nowadays to endorse the concept of “relational autonomy” (Gómez-Vírseda et al., 2020). According to the latter, people are not solipsistic agents but are defined by their relationships and depend on “significant others” to make decisions. This way, caregivers might often influence a patient's decision-making process regarding medical treatments, bringing with them a range of emotional reactions, interpersonal dynamics, and expectations (Hobbs et al., 2015; Laidsaar-Powell et al., 2016; Shin et al., 2016; Longacre et al., 2018).

Regardless the evidence about the effects that patient-caregiver interaction may have at both a psychological and decision-making levels, few studies assessed the effectiveness of psychosocial interventions, aimed at helping caregivers to be more involved in the treatment decision-making (TDM) process (Garvelink et al., 2016). Other studies focused on the effectiveness of dyadic psychological intervention on distress management (e.g., anxiety, depression, hopelessness, etc.) and patients' Quality of Life improvement (Regan et al., 2012; Griffin et al., 2014; Hu et al., 2019). The contents of psychological intervention protocols were psychoeducational, skills training or therapeutic counseling (e.g., anxiety, depression, hopelessness, etc.). These interventions could be either couple-based interventions involving both the patient and the caregiver in the same session, or interventions offered to the caregiver only, or to the caregiver and the patient independently in separate sessions (Hu et al., 2019).

Drawing upon this background, this study protocol aims to firstly evaluate the efficacy of a dyadic psychological support consultation for newly diagnosed breast and prostate cancer patients-caregivers on their level of agreement related to TDM, through a randomized research design. We also want to estimate related improvement in patient' compliance/adherence to treatments, together with the satisfaction with the (shared) medical decisions, and their psychological distress. We focus on breast and prostate cancer patients not only for a pragmatic reason, being the two most common disease in women and men, respectively, but even because they can have an early detection, so that we can explore caregivers' involvement from the diagnosis onward (Northouse et al., 2007; Dorros et al., 2010; Segrin and Badger, 2010; Oliveri et al., 2020b). Moreover, breast and prostate cancers belong to those types of cancers where caregivers' involvement can be necessary, since some decisions for therapies also involve the sphere of intimacy and fertility (Northouse et al., 2007; Dorros et al., 2010). In these cases, partners ideally play a fundamental role in making decisions, insofar as the decision has an impact on them as well (Segrin and Badger, 2010; Muzzatti et al., 2020; Cincidda et al., 2022). For this reason, the promotion of specific interventions to support dyads facing the diagnosis of breast or prostate cancer becomes even more crucial.

Accordingly, this study presents the following interrelated hypotheses:

a) The psychological support consultation will be effective in increasing the alignment between newly diagnosed breast and prostate cancer patients and their caregivers, regarding treatment decisions and in reducing or managing the possible disagreement/conflict that may arise among them.
b) The psychological support consultation will improve newly diagnosed breast and prostate cancer patients' treatment adherence, satisfaction with TDM and their psychological distress.

## Methods and analysis

### Study aims

The *primary aim* of the present study is to assess whether a psychological support intervention for newly diagnosed breast and prostate cancer patients-caregivers' dyads may promote their synergy in the TDM process and may improve the compliance/adherence to treatment and the satisfaction with the (shared) medical decisions through a randomized study.

The *secondary aim* are: (a) to assess the preferences and degree of agreement between newly diagnosed breast and prostate cancer patient and caregiver on the latter's involvement in the TDM process; (b) to investigate which factors and dynamics contribute to a collaborative relationship between newly diagnosed breast and prostate cancer patient and caregiver, and determine the level of involvement of the caregiver; (c) to understand what are the effects that the involvement of caregivers have on newly diagnosed breast and prostate cancer patients in terms of patients' perceived social support, satisfaction with the clinical path, compliance with therapy, quality of life, anxiety and depression, and perceived empathic relation with clinicians.

Finally, since the issue of caregiver's involvement has been also investigated within the bioethical literature and presents bioethical connotations as well, the ethical issues surrounding this dyadic relationship will be also explored. Accordingly, in parallel with the main study, a secondary investigation exploring the presence and nature of ethical disagreements within family relationships, in the context of diagnosis of and treatment to cancer will be carried out.

### Study design and setting

This study will be conducted at the European Institute of Oncology of Milan (Italy). The IEO is a specialized Hospital and internationally recognized Cancer Center located in Italy working on research, prevention, diagnosis, and treatment of cancer. The IEO offers to the patients innovative and personalized care, from the diagnosis onward. Study design is longitudinal randomized, with a 1:1 allocation ration analyzing the proposed primary and secondary aims.

Participants will be randomly assigned to one of the two following arms: (1) dyads receiving a psychological support consultation (experimental group); (2) dyads receiving no psychological support consultation (control group). For ethical reasons, the dyads in the control group will be offered the option to receive the psychological support consultation at the end of the study, but only in case the psychological support consultation turns out to be beneficial for the dyads. Indeed, the study did not foresee a cross sectional design.

The dyads assigned to the experimental group will be contacted by the clinical psychologist to schedule an appointment for the psychological support consultation. The consultation will be performed within the period of diagnostic assessment, preferably within the date of pre-hospitalization for surgery or for starting other cancer treatments. The consultation will be carried out by a clinical psychologist with a master's degree in clinical psychology, a state certificate exam, and previous internship in psychological support intervention for cancer patients.

The consultation will last 60 min and will include the following steps and areas of focus:

- the psychologist will open the consultation by asking the dyads if they would like to narrate their emotional reaction to the cancer diagnosis, if they were together when they got the bad news or if the patients later communicated diagnosis to the caregivers. This way, the psychologist can start sharing their emotional state of the dyads and their communication style;
- then, the psychologist will assess the dyad's preferences about their mutual involvement in the clinical assessment path (e.g., participating together in medical examinations) and in the decision-making process, and their level of agreement regarding the ideal care path. The psychologist can help dyads to explicit their emotional status, have an effective and assertive exchange of ideas, provide different input on how to handle the upcoming star of treatments. Moreover, the psychologist will evaluate their feelings and perception about the relationship with the medical team to assess whether a therapeutic alliance between patients, caregivers and clinicians can be structured.
- finally, the psychologist will help them in reporting their emotions/fears and mutual needs, to help the dyad go through this difficult moment of life together providing adequate support to each other. During the consultation, the psychologist will be available to address even more topics of interest to the dyad to favor the care path and a better quality of life, such as how to behave with minor children or with extended family.

The psychologist who is going to carry out the consultations will be supervised by a coordinator with a specialization in psychotherapy, with the aim of solving any doubts.

**Figure 1:**
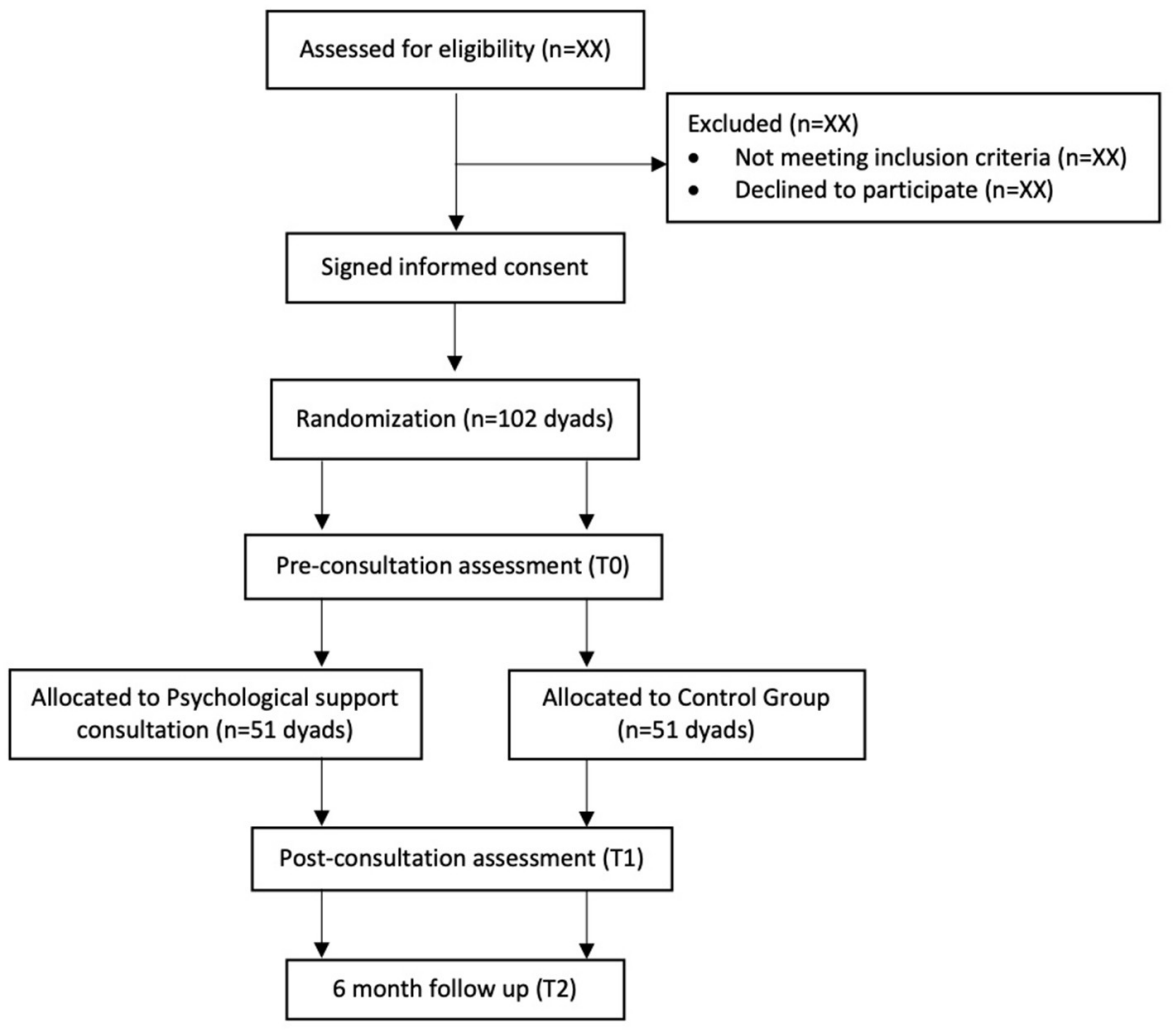
Study protocol flowchart.

The evaluation through a set of standardized questionnaire swill be carried out at three time points: initial evaluation before randomization (baseline, pre-consultation; T0), after the pre-hospitalization (and post psychological consultation for the experimental group; T1), at 6 months after the beginning of the medical treatment (T2). Between the baseline evaluation (T0) and the second evaluation (T1), the experimental group will meet the psychologist for the consultation and the control group will not receive any intervention, but both groups will have made decision about cancer treatments with their referring oncologists. The average time between the two assessment T0 and T1 is about 4 weeks (see Figure 1 for the study flowchart).

The assignment to one of the two arms will not be disclosed to the participants and to the experimenters who will interface with the subjects for consultation and psychological assessment through questionnaires. Only personnel who analyze the data collected from the study will be aware of participants who receive psychological support consultation. Participants will be randomized during T0 assessment, immediately after the collection of socio-demographic and clinical data and psychological measures.

## Measures

### Time 0 assessment

Before the randomization, all the dyads will fill out a set of questionnaires separately, administered electronically *via* the Qualtrics™ online platform. A specific link to the survey will be created and provided *via* email or mobile phone to the personal email or telephone number of the patients and the caregivers. In the mail text, the researcher reminded the patients and caregivers to fill out the survey autonomously stressing the importance of answering in a personal and sincere way. The survey will take about 30 min to complete (assessed in a pilot with 5 dyads) and will include the following domains and related measures:

#### Demographic

Gender, age, education, occupation, origin, degree of relationship with the patient or caregiver.

#### Decisional role preference

A modified version of the Control Preference Scale questionnaire (Degner et al., 1997) is administered to examine patients' and caregivers' preference for caregiver involvement in cancer TDM, as used in other studies (Giordano et al., 2008; Shin et al., 2013, 2017, 2018). One item evaluates patients' and caregivers' desirable level of caregivers' involvement in TDM. Response options are 5 that refer to an active, collaborative, or passive role in TDM.

#### Dyadic communication

Five items from the Personal Assessment of Intimacy in Relationships scale [PAIR (Schaefer and Olson, 1981)] will be included to evaluate dyadic communication (3 items from the emotional intimacy subscale and 2 items from the intellectual intimacy subscale). These items are measured on a 5-point Likert scale, from 1 = does not describe me/my relationship at all to 5 = describes me/my relationship very well. Scores can be interpreted in terms of the difference between the dyads' scores. Every participant can decide for him/her-self what is good or ideal. The internal reliability coefficient (Cronbach α) of the communication subscale of PAIR is 0.80 (Constant).

#### Coping style

The Miller Behavioral Style Scale [MBSS (Miller, 1987)] is a coping style measure used to determine the information-seeking behaviors of individuals under threat. It is composed of 32 items and aims at differentiating between individuals who actively seek threat-relevant information (monitoring style) and those who distract themselves from threatening information (blunting style). Four imaginary stress evoking situations are described (i.e., a dentist, hostage, redundancy, and airplane scenario) and participants can choose all responses (8 responses) that they think are most characteristic of how they would act in each situation. Four responses referred to the monitoring style and four to the blunting style. This psychological variable can be considered a trait variable that remains stable over time. Cronbach's α coefficients for the monitoring and blunting sub-scales were 0.65 and 0.41, respectively (Rees and Bath, 2000).

#### Anxiety and depression

The Hospital Anxiety and Depression Scale [HADS (Zigmond and Snaith, 1983)] is a self-administered measure used to screen for the presence of anxiety and depression. It consists of 14 items on a person's mood in the past week. Seven items assess depression, referring to anhedonia (an inability to experience pleasure) and to the appearance and feelings of slowing down. Seven items assess anxiety, both autonomic anxiety (panic and butterflies in the stomach), and tension and restlessness (Dunbar et al., 2000). Each item is rated on a 4-point scale for a total score ranging from 0 to 21 for each subscale. A higher score indicates higher distress and the cut-off points for establishing the presence of anxiety and depression is set at 8. This scale has been adapted and validated into Italian both for cancer patients and a community sample (Annunziata et al., 2011; Iani et al., 2014).

#### Perceived social support

The Multidimensional Scale of Perceived Social Support [MSPSS (Zimet et al., 1988)] consists of 12 items measuring the perceived adequacy of social support from three factors: Family, Friends, and Significant Others. The respondents are asked to indicate their level of agreement to each item on a seven-point Likert scale (1 = strongly disagree to 7 = strongly agree). This scale has been adapted and validated in Italian by Di Fabio and Palazzeschi (2015), showing an internal consistency (Cronbach's α coefficients) of 0.89 (Fabio and Busoni, 2009).

#### Health status

The SF-12 Health Survey is a shortened version of the SF-36, created to reduce the burden of response (Ware et al., 1996; Jenkinson et al., 1997; Gandek et al., 1998). The SF-12 is a self-reported outcome measure assessing the impact of health on an individual's everyday life and it is often used as a quality-of-life measure. It is composed of 12 items, extracted from the SF-36, and related to the same domains of SF-36. In particular, the following domains are evaluated by two items: limitations in physical activities because of health problems; limitations in social activities because of physical or emotional problems; limitations in usual role activities because of physical health problems and limitations in usual role activities because of emotional problems. Instead, bodily pain, general mental health (psychological distress and wellbeing), vitality (energy and fatigue) and general health perceptions are evaluated by one item. Subjects are asked to answer by evaluating the day they completed the questionnaire and the previous 4 weeks. The scoring provides two summary measures related to physical and mental health (PCS-12 and MCS-12). Kodraliu et al. (2001) assessed the SF-12 in various Italian settings, including the general population and specific patient groups, showing that the SF-12 has good validity.

#### Shared decision-making

The Shared Decision-Making Questionnaire (SDM-Q-9) was developed in a theory-driven manner and measures the extent to which patients are involved in the process of decision-making from the perspective of the patient [patient version SDM-Q-9 (Kriston et al., 2010)]. It is composed of 9 items/statements, which can be rated on a six-point Likert scale from “completely disagree” (0) to “completely agree” (5). The questionnaire will be adapted for the caregivers involved in the following study. The SDM-Q-9 was translated into English and Italian, allowing for use in international research (Kriston et al., 2010). The questionnaire was validated in a psychiatric clinical sample showing a Cronbach's α coefficient of 0.86 (de Filippis et al., 2022).

#### Empathy toward physicians

The Consultation and Relational Empathy (CARE) Measure (Mercer, 2004) is a person-centered process measure composed of 10 items. It measures empathy in the context of the therapeutic relationship during a one-on-one consultation between a clinician and a patient. Participants are asked to evaluate how the doctor was in some situations on a six-point Likert scale, ranging from poor (0) to excellent (5) or does not apply (6). The tool will be adapted for the caregivers involved in the following study. The Italian version of the CARE measure showed high internal reliability (Cronbach's α = 0.962) (Natali et al., 2022).

### T1 measures

#### Decisional role preference

An original modified version of the Control Preference Scale questionnaire (Degner et al., 1997) is administered estimating the actual level of caregivers' involvement in TDM from patients and caregivers' perspectives.

#### Decisional role

An Italian translation of original questions drafted by Shin et al. (2017) are included to investigate the patient's perceived benefit/harm of caregiver's involvement in TDM. First, respondents were asked to rate the level of family involvement regarding communication, treatment decisions, and psychological support on a 3-point Likert scale: harmful, neither harmful nor helpful, or helpful (Original items were: “family involvement is—harmful, neither harmful nor helpful, helpful—for communication, treatment decision, psychological support”). Then, respondents were also asked to indicate their level of agreement on a 4-point Likert scale (from 1 = strongly disagree to 4 = strongly agree) with the following statements regarding family involvement in cancer TDM: “It hampers patient autonomy,” “it complicates the cancer TDM process,” and “it leads to a harmonious decision,” and “Families have the right to be involved.”

#### Satisfaction with decisions

The Satisfaction with Decision scale (SWD) measures the satisfaction with health care decisions (Holmes-Rovner et al., 1996). It consists of 6 items rated on a five-point Likert scale from “strongly disagree” (1) to “strongly agree” (5). The tool will be adapted for the caregivers involved in the following study. The scale has excellent reliability (Cronbach's alpha = 0.86).

The following questionnaires administered at T0 will be also included also at T1, to evaluate the change in the psychological status: The Hospital Anxiety and Depression Scale [HADS (Zigmond and Snaith, 1983)]; The Multidimensional Scale of Perceived Social Support [MSPSS (Zimet et al., 1988)]; The SF-12 Health Survey (Ware et al., 1996); The 9-item Shared Decision-Making Questionnaire [SDM-Q-9 (Kriston et al., 2010)]; The Consultation and Relational Empathy (CARE) Measure (Mercer, 2004).

### T2 measures

#### Adherence to therapies

The Adherence Determinants Questionnaire [ADQ (DiMatteo et al., 1993)] is a multifactorial tool aiming at evaluating the elements of patients' self-adherence to cancer treatments, taking into consideration a set of cognitive and motivational skills, as well as social and behavioral variables. Response options for each item comprise 5-point Likert scales (1 = *strongly disagree;* 2 = *disagree;* 3 = *neither agree nor disagree;* 4 = *agree; 5* = *strongly agree*). Seven subscales are considered: Interpersonal Aspects of Care; Perceived Utility (Benefits/Costs and Efficacy); Perceived Severity; Perceived Susceptibility; Subjective Norms; Intentions; Support/Barriers. The components of the ADQ were found to be generally reliable (median alpha reliability = 0.76) (DiMatteo et al., 1993).

In addition, the following questionnaires will be re-administered: item from Shin and colleagues (Shin et al., 2017) for Decisional Role; 5 items from PAIR (Schaefer and Olson, 1981) (dyadic communication); The HADS (Zigmond and Snaith, 1983); The MSPSS (Zimet et al., 1988); The SF-12 Health Survey (Ware et al., 1996); The SWD (Holmes-Rovner et al., 1996).

### Empirical bioethics study

In parallel with the main study, an empirical bioethics study will be carried out (Borry et al., 2005; Dunn et al., 2012). Drawing from a systematic review exploring the nature of conflicts within dyads from an ethical standpoint (Cincidda et al., ongoing work), namely, what we refer to as “moral conflict”, conflict between patient and caregiver may arise when the latter is involved in the patient's care path (Levine and Zuckerman, 1999, 2000; Vivian, 2006; Kramer et al., 2010; Hauke et al., 2011; Boelk and Kramer, 2012; Shin et al., 2016; Longacre et al., 2018; Benson et al., 2019; Hansen and Tjørnhøj-Thomsen, 2020). The concept of conflict can be analyzed both from a psychological and bioethical perspectives, depending on whether the conflict presents a psychological or ethical connotation. Current bioethical literature on the topic, has shown that “moral conflict” between parties may originate because of different factors. A first source of “moral conflict” lies in the so called “ethical disagreement”, namely the fact that the patient and the caregiver have different (and in some cases incompatible) values with respect to care decisions and treatment goals (Hauke et al., 2011; Kramer and Yonker, 2011; Korfage et al., 2013; Benson et al., 2019; Laryionava et al., 2021). In other cases, “moral conflict” arises when the patient or caregiver base their decisions on personal interests, creating what we referred to as “conflict of interest” (Levine and Zuckerman, 1999, 2000; Blackler, 2016; Laryionava et al., 2018). Other authors reveal that “moral conflict” can be related to the concept of agency. Indeed, during the oncological care path, patients and caregivers are *autonomous agents* that should make medical decisions. Conflicts may arise in this case when patients and/or caregivers feel a compromising of their autonomy, experiencing moral distress. Caregivers may unintentionally manipulate patients, compromising their autonomy, or viceversa, patients may oblige caregivers to decide in place of them, transferring their autonomy (Vivian, 2006; Blackler, 2016; Osamor and Grady, 2018; Benson et al., 2019). Finally, moral conflict may be the consequence of purely relational and experiential aspects. This last kind of moral conflict originates from a non-recognition of the other as “ontologically different” from the subject primarily asked to take the decision. Sometimes, cancer patients don't feel recognized in their ontological individuality by their caregivers, even though they appreciate their support (Kagawa-Singer and Wellisch, 2003; Hansen and Tjørnhøj-Thomsen, 2020).

Drawing upon these premises, a parallel empirical bioethics study will be conducted, with the aim to further explore the concept of moral conflict, investigating it in a qualitative manner. Differently from most of the bioethics literature which focuses on late cancer, i.e., when the patient is proxy to death, our study aims to investigate this phenomenon at the beginning of cancer trajectory, when potential moral conflicts may impact throughout the entire therapeutic process (Davies et al., 2015). A group of 20–25 dyads already enrolled for the main study will be invited to reply to in-depth qualitative face-to-face semi-structured interviews aimed at identifying and mapping possible decision-making conflicts of a moral nature, namely “moral conflict”, arising in the patient-caregiver relationship and potentially affecting the care process. After a very brief explanation of what an ethical issue is and to what extent it differentiates from, e.g., psychological issues, dyads will be asked whether they have already experienced some sort of moral conflict, to describe its nature, and to report whether and how this conflict impacted on their relationship, medical decisions, and adherence to cure. Furthermore, it will be investigated whether dyads would consider useful/beneficial to be offered for free a support service dedicated to the discussion and management of moral conflicts occurring within the patient-caregiver relationship and potentially affecting the oncological care process.

### Participants

Patients who had access to the Division of Breast Surgery and to the Division of Urologic Cancer Surgery at the European Institute of Oncology (IEO), with a suspected cancer or a newly cancer diagnosis and accompanied by a caregiver, will be reported by the oncology and nursing team. Then, the researcher will contact the reported patients though a phone call and/or an email. During the first contact, one researcher of the team informs patients about the study purposes and the procedure. Then, patients are asked to share the information with a caregiver (the definition of caregiver will be provided so that the patient can consciously decide about their main caregiver) and to invite him/her to join the study. An official invitation letter, the informed consent, and the link to the online survey (*via* Qualtrics™ Platform), will be then sent by email to the enrolled patients and referred caregivers. Time schedule of recruitment, interventions, assessment, and follow-ups are available in the flow diagram (see Table 1).

**Table 1:** Schedule of enrolment, intervention, and assessment.

	**Enrolment**	**Allocation**	**Post-allocation**	**Close-out**
Timepoint		T0	T1	T2
**Enrolment**				
Eligibility screen	X			
Informed consent	X			
Allocation		X		
**Intervention**				
Psychological support consultation		X	X	X
Control group		X	X	X
**Assessment**				
Sociodemographic data		X		
CPS		X	X	
Decisional role			X	X
PAIR-communication subscale		X		X
MBSS		X		
HADS		X	X	X
MSPSS		X	X	X
SF-12		X	X	X
SDM-Q-9		X	X	
CARE		X	X	
SWD			X	X
ADQ				X

### Eligibility criteria

Tables 2, 3 show inclusion and exclusion criteria for newly diagnosed breast and prostate cancer patients and for the caregivers. Regarding caregivers, we have decided to include any person that the patients considered important to them and that they felt by their side in this difficult period.

**Table 2:** Inclusion and exclusion criteria for patients.

**Inclusion criteria**	**Exclusion criteria**
Patients with a newly breast or prostate cancer diagnosis that currently are within the diagnostic assessment process and have to discuss their treatment with the oncologists.	Presence of early mental disorders (before age 40) or severe neurological disorder.
Early-stage cancer (I or II)	Patients with advanced stage cancer for which the path is already defined (palliative care patients).
Age ≥ 18	
Able to give informed consent^a^	
Able to read, speak, and understand Italian^a^	

a This criterion was not present in the study protocol approved by the Ethical Committee.

**Table 3:** Inclusion and exclusion criteria for caregivers.

**Inclusion criteria**	**Exclusion criteria**
Taking care of the patient	Presence of early mental disorders (before age 40) or severe neurological disorders
Age ≥ 18	
Able to give informed consent^a^	
Able to read, speak, and understand Italian^a^	

a This criterion was not present in the study protocol approved by the Ethical Committee.

### Sample size and power calculation

One hundred two dyads are needed for this study considering 51 male patients enrolled in the Division of Urologic Cancer Surgery and 51 female patients enrolled in the Division of Breast Surgery at IEO in Milan.

The variable “compliance with therapy” measured by ADQ (DiMatteo et al., 1993), including the subscales Interpersonal Aspects of Care (range: from 8 to 40) and Subjective Norms (range: from −18 to 18), was used as the primary outcome of interest in the sample size calculation. The hypothesis is that the involvement of caregivers increases the patient's adherence to treatment recommendations, which in turn directly impacts the patients' prognosis as widely demonstrated in the literature (Ricci-Cabello et al., 2020). The sample size calculation was based upon the assumption that we will observe a difference of 3 points in the average values of the two subscales IAC and SN, in favor of the intervention group. A standard deviation of ~5 points is assumed for both subscales (untransformed values) according to data presented by DiMatteo et al. (1993), leading to a hypothesized effect size of 0.6. This effect size should be considered as a medium-large effect according to Cohen (2013), and it is considered to provide clinical relevance. A two-sample *t*-test will be used to compare the mean subscale values in the intervention group vs. control group. With a 1:1 randomization ratio, a total of 88 patients (44 in the intervention group and the 44 in the control group) are required to provide 80% power to detect a difference ≥ 3 points, with a type I error of 5% (two-sided *t*-test). Assuming a drop-out rate at 6-months of ~15%, a total of 102 patients will be required.

### Data analyses

Missing data: presence of missing data will be checked before conducting the main analyses. If a subject does not complete T1 and T2 he/she will be excluded from the analysis.

Normal distribution of continuous variables will be assessed (using skewness and kurtosis) before conducting the main analyses. Non-normal distribution will be handled by applying either non-parametric tests or SEM with an estimator for non-normal distribution.

Statistical analyses will be performed in SPSS version 25. Socio-demographic data collected in this study will be described in terms of mean, standard deviation, median, minimum, and maximum or reported frequencies in combination with confidence intervals. The preliminary analysis will be carried out through the Pearson or Spearman correlations r between the variables considered and by exploratory analysis of variance. To compare the two experimental and control groups, the *t*-tests (in case of normal distribution) or Mann-Whitney U tests (in case of non-existence of a normal distribution) will be applied, or cross tables with the chi-squared tests and (if necessary) the generalized exact Fisher's tests.

The main analysis will consist of an evaluation at different time-points of the psychometric scales, using linear models with mixed effects for repeated measures. The scales of interest will constitute the dependent variable, whereas the independent fixed factors will be the time of visit, the intervention group, the scale measured at baseline and the interaction term between the time of the visit and the intervention and, as a random effect, the patient. Multiple linear regressions will be calculated for both groups to evaluate the weight of each variable on the main outcome.

The semi-structured interviews will be analyzed by NVivo v10, a qualitative data analysis software.

## Discussion

People who take an active role in their health have a greater feeling of control and are more likely to adopt positive health behaviors (Arnaboldi et al., 2017; Oliveri et al., 2020a, 2022; Ongaro et al., 2022). For this reason, patient empowerment is more important than ever to manage a serious condition like cancer: patients are often called to make decisions together with their oncologists about the treatment to undergo, favoring the shared decision making (Howell et al., 2021). However, patients are often accompanied by at least one caregiver, who play a fundamental role in the shared decision-making process.

The role of caregiver in the shared decision making is not yet clear: sometimes he/she can be a facilitator, other times he/she can be a barrier to shared decision making. For that reason, the present research intends to verify whether a psychological support intervention can improve the triadic communication between patient, caregiver, and physician, and consequently the patient's adherence to treatments and the satisfaction with medical decisions.

We expect a positive impact of such intervention on patient's anxiety, depression and distress, and improvement in the perceived social support. Furthermore, this study would like to evaluate whether the patient's preferences on the involvement of the caregiver during the oncological care path depend on relational dynamics and/or on individual psychological variables.

Moreover, through this study we would explore if the presence of disagreements between patient and caregiver can affect the patient's cancer care pathway, both in terms of both in terms of processing the information received from the doctors and the adherence to therapies. Finally, the present study aims to investigate the ethical consequences that might emerge from the involvement of the caregiver. For these purposes, a concurrent longitudinal investigation will be performed.

Data gathered from this study may inform health care providers, policy makers, and public health managers about the caregiver's involvement and how to regulate it.

The *potential impact* of this project is also to start developing a specific intervention protocol to promote a functional caregivers' involvement in cancer care pathway, improve patient's wellbeing, the interaction with physicians and the patient's compliance with the cancer treatment.

Notwithstanding, it is necessary to mention some of the limitations that can be linked to this study. First, the inclusion of caregivers with different relationships with patients (all possible dyads are included) can create non-homogeneity in the sample composition, as there may be specific relationship factors that influence the results. In addition, there may be a large loss of participants in the control group, that might not see any direct advantage in participating, so it may be strategic to remind them that they may require hospital-provided psychological support at the end of the study (albeit for a fee).

Despite these limitations, the results that are expected to be obtained after conducting this trial may be useful in helping patients-caregivers' dyads during the cancer care path to reduce possible disagreement within the dyads and enhance patients' adherence to cure and the satisfaction with the decision made and more in general to better their daily life.

## Ethics statement

The present study is compliant with the recommendations set forth in the Helsinki Declaration (World Medical Association, 2013) and the CIOMS Guidelines (Bandewar, 2017), as well as with the principles of biomedical ethics reported in the Belmont Report (Department of Health, Education, and Welfare and National Commission for the Protection of Human Subjects of Biomedical and Behavioral Research, 2014). This study presents a fair balance between risks and benefits, both for study participants and for future patients affected by the same condition of enrolled patients. Since the study is not a clinical trial, no physical risks directly related to the participation in the research are expected. Although psychological risks are not expected too, in case these raised from participation, psychologists responsible for the study will promptly intervene and take care of the patient. In particular, the reference psychologist can take charge of the patient or caregiver by proposing a psychological support intervention or send them to colleagues of the division for a psychotherapy intervention or otherwise send them to colleagues in the patient's or caregiver's territory of origin. Regarding the benefits, this study can improve patients and caregiver's awareness of their own decisional and psychological state. The dyads enrolled in the experimental group will benefit from psychological support that may hopefully improve the relational synergy, the communication processes and, more generally, the relationship between the two. Psychological support will also help the dyad to get to know each other better both in aspects related to the decision-making process of care and to the needs of both. Finally, the study will enable the development of a personalized psychological decision support system that will hopefully help both enrolled and future patients to better cope with the disagreement that arises within the dyads and have a more shared decision-making process. All this will make it possible to leverage the increasing need for a multidisciplinary team for patients entering the hospital. The principle of self-determination is also respected. A devoted informed consent form will be signed by participants before participation. Sign on the informed consent form will be proceeded by a dialogic consent process necessary to ensure informed voluntary and aware participation in research. Regarding respect for privacy, the study will be carried out according to the General Data Protection Regulation, Regulation (EU) 2016/679 (Holmes-Rovner et al., 1996). All data will be collected in a pseudo-anonymized form. As to the main study, data will be analyzed in an anonymous manner, whereas as to the empirical bioethics study these will be analyzed in pseudo-anonymized form. Data will be treated confidentially and used only by the collaborators in the present study for scientific purposes related to what stated in the research protocol. Ethical approval has been obtained for this study from the Local Research Ethics Committee of the IEO (Approval Number: R1598/21-IEO 1702).

## Author contributions

CC conceptualized the ideas, made up the entire protocol and wrote the first draft in collaboration with VS and SO. CC, SO, and VS wrote, reviewed, and edited the manuscript. GP contributed with important intellectual content and supervised the whole process. All authors contributed to the article and approved the submitted version.

## Funding

The present work was partially supported by the Italian Ministry of Health with Ricerca Corrente and 5 × 1000 funds for IEO European Institute of Oncology IRCCS.

## Conflict of interest

The authors declare that the research was conducted in the absence of any commercial or financial relationships that could be construed as a potential conflict of interest.

## Publisher's note

All claims expressed in this article are solely those of the authors and do not necessarily represent those of their affiliated organizations, or those of the publisher, the editors and the reviewers. Any product that may be evaluated in this article, or claim that may be made by its manufacturer, is not guaranteed or endorsed by the publisher.
